# The Treatment of Post-Nasal Catarrh

**Published:** 1879-12

**Authors:** Wm. R. D. Blackwood


### THE TREATMENT OF POST-NASAL CATARRH.

Read before the Philadelphia County Medical Society, September 24, 1879.
By WM. R. D. BLACKWOOD, M.D.

Under the title of our subject this evening may properly be included the treatment of an extensive series of disorders of the naso-pharyngeal space, evidencing all grades of severity, from the simplest chronic coryza, of little account, to the formidable œzena, which, in its destructive ravages, sometimes threatens, and occasionally destroys, life itself.

The great majority of cases of catarrh are not under medical treatment of any kind, and the majority of the remainder are in the hands of advertising philanthropists, retired clergymen, Indian, botanic, eclectic, homœopathic, and such like illiterate quacks. Thousands of cases have been treated by eminent and by skillful men, without either cure or appreciable relief. Sweeping as this assertiom may seem, let gentlemen only interrogate families under their charge, many of whose members suffer from catarrh without applying for advice, and they will readily discover its truth. The natural outcome is that post-nasal catarrh is considered an incurable disease, and, as the laity is not to blame for its mistaken opinion, would it not be well that an effort be made to undo this error by paying the subject more attention than it apparently receives, and which, from its importance, it undoubtedly deserves? It is singular that so much study has been given to some diseases and so little to others, and, as it does not always follow that the gravity of an affection determines the interest felt in its investigation or treatment, it is possible that in this way the subject under consideration may have been unworthily neglected.

Very little inquiry will elicit the reason why so little is successfully done in the treatment of catarrh. Briefly stated, the failure consists in relying upon general constitutional treatment, which is all well enough as an auxiliary, but not sufficient in itself. To cure the sufferer demands the persistent, skillful, personal attention of his medical adviser, and unless this course is strictly and conscientiously followed, the patient will receive little benefit and his physician only discredit.

The disagreeable nature of the service, the time and trouble involved, and the difficulty in some cases of mechanical treatment, have deterred the larger part of the profession from devoting the requisite personal attention to their patients, and the expense attendant upon a necessarily extended course in the hands of most specialists has prevented many from receiving the benefits which would accrue from their services.

I do not intend speaking of the pathology of naso-pharyngeal catarrh, there being too much room for difference of theory, and too little time for discussion. The vital point is the treatment of our patient. What is to be done? No claim of novelty is intended in this paper, but it may be that suggestions thrown out as the result of successful management might be elaborated by others, and serve a useful end to those who have hitherto given the question less attention than it of right deserves.

First, then, as to mechanical treatment—an essential in all cases. The instruments requisite are few and simple in construction and application. At their head stands the nasal douche, an invaluable and indispensable assistant, not to be replaced by any other. The objections to its use are avoidable by simple precautions. Employ water always at a temperature of 100° F.; add one drachm of common table salt to each pint of water used; do not permit the patient to swallow during the flow of the douche; hold the reservoir no higher than the eye of the sufferer; incline the head slightly forward over a basin; use simple douche, free from valves or complicated mechanism; an ordinary tin or glass funnel, with a yard of quarter-inch rubber tubing and a conical glass, wood, or porcelain nozzle, is as good as any, is cheap, durable, and easily made. Do not trust the douche in the hands of the patient until its use is thoroughly understood and accurately managed. In stupid, perverse, or nervous persons, never trust it to them; always apply it yourself. I have never had any trouble occur through the use of the douche. The posterior-nares syringe is a valuable adjunct, as also is the atomizer, in getting at the vault of the pharynx, which they do in many cases much more effectually than does the anterior douche. Although, theoretically, the steam atomizer appears preferable, because of the temperature being readily maintained, yet the difficulty of directing the spray in other than a horizontal line, and the view of the parts being intercepted by the boiler, render the hand-ball apparatus more convenient, especially in conjunction with simple adjustable tubes, which afford a lateral or vertical discharge at pleasure. A convenient though expensive apparatus is the compressed-air mechanism of Codman & Shurtleff, by means of which a steady flow may be maintained for any desired length of time. In common with those applied by the douche, all atomized solutions should be at blood heat and of sufficient density. Caustic and sponge-holders, brushes of various sizes, an insufflator, a tenaculum for the uvula, a tongue-depressor, an anterior-nares speculum, and a rhinoscopic mirror are requisite, with a strong light, natural or artificial, in the management of which nine-tenths of the imposing array of mechanism usually encountered may be dispensed with. Each patient should have his own douche, and all instruments employed in office practice must be kept thoroughly clean. Scrupulous care is to be observed in syphilitic cases that the ordinary catarrh of one patient is not complicated by specific inoculation from another.

In the department of materia medica the list of necessary agents will vary with the taste of the physician, some drugs developing powers in the disease under consideration, as in others, according to the ability shown in their use or the idiosyncrasy of each particular case. In my practice the list is not extensive. The first point in treatment is the thorough cleansing of the parts at least twice daily, the ordinary solution of sodium chloride being a satisfactory one. Occasional alternations with a solution of potassium biborate, which I prefer to the sodium biborate, in similar proportion, will prove effectual where the secretion is free and the posterior nares blocked. The addition of from three to five grains of potassium permanganate in solution, when the wash is from one-half to three-quarters expended, will modify the retor and render the subsequent steps much more comfortable to the medical attendant. The liquor sodæ chlorinatæ replaces the permanganate satisfactorily, especially in delicate blondes. Potassic chlorate may also be added, but it is serviceable only from its local effect, no constitutional impression being apparent through it in my hands. The use of from one-half to one ounce of the distilled extract of witch-hazel, as prepared by Mr. McKelway, of this city, is frequently efficacious, but the ordinary fluid extract made by displacement is not reliable. The simple or compound tincture of benzoin is an admirable remedy, both locally and internally. The quantity of water necessary is determined by the amount of offensive secretion, and varies from one pint to many, the prime object being the removal of all crusts, pus, mucus, or blood, without which subsequent medication must fail from the remedy not reaching the congested or abraded tissue. In mild or recent cases the careful cleansing, as described, of the parts will sometimes, if prolonged, effect a cure; but such success is uncommon in this intractable disease.

In long-standing and stubborn cases, after preparing the parts as suggested, a number of astringent or alterative remedies can be selected. That chosen may be employed either in solution, or, as is sometimes more efficacious, in dry powder projected on the parts by an insufflator. Snuffing the powder from the hand does not act so accurately, in consequence of much of the medicament being either detained anteriorly by the turbinated bones, or, having passed them, being drawn into the lower pharynx. Alum, bismuth, cubebs, tannic or gallic acid, with, if thought proper, ferric, cupric, or zincic sulphate, will act admirably in many cases. Granulations or adenoid tissue, if excessive, may be removed, when accessible, by the curette or galvano-cautery. I have not observed distinct patches of ulceration as often as the literature of the subject would imply their existence, except in caries or necrosis of the bones of the nasal passages (or flues, as a locomotive-building patient of mine aptly interprets them), any apparent abrasion being generally diffused; but should examination detect areas thus affected they should be stimulated or cauterized, as required, by strong solutions or the solid silver nitrate. Glacial acetic acid is a favorite with me in such instances, and tincture of iodine frequently acts admirably. Loose portions of bone or easily-detached pieces should be carefully removed, and a very small spicula will, if overlooked, be often productive of continuous discharge. I have not performed Rouge's operation in any case of catarrh, but some time ago, in assisting a friend in an operation on the upper maxillary, the feasibility of reaching the nares by that method was very apparent, and in a second operation on the same patient by myself I removed another part of the superior maxillary and a portion of the vomer, without dividing the upper lip or cheek, as had been done in the previous operation. The rapidity of union without any suppuration was remarkable, and the freedom from scar or deformity by this method is invaluable.

Attention to the general health is of moment. The secretions of the alimentary canal, the kidneys, and the skin should be inquired into, and placed in proper condition if defective. I have found the Turkish bath a most efficient auxilliary in all stubborn cases. The constitutional remedies employed will vary as indicated by apparent dyscrasia. Mercuric bichloride is notably a tonic and alterative in many cases, even were syphilitic complication is not evident. I usually combine the alterative selected with compound fluid extract of stillingia, in drachm doses, three or four times daily. When well borne, copaiba, long continued, is of great service. Ferric iodide, with or without potassium iodide, and sometimes calcium chloride, are excellent in chronic scrofulous cases. Iodoform has not been of much use in my hands, either locally or constitutionally, nor has carbolic acid. As to salicylic acid or its salts I cannot yet form an opinion of their merit, although as a detergent the sodium salicylate is apparently good.

Of all therapeutic remedies I value none more highly than electricity, which convinced me of its value through the importunities of a patient whom I was treating at the time for myalgia, and who suffered badly from post-nasal catarrh. He insisted that electricity would cure his catarrh; and so it did, contrary to my opinion. It cannot, of course, be relied on in every case, but it is a valuable adjunct. Either the galvanic or the faradic current may be necessary,—sometimes both,—but the induced current appears to be the most generally applicable, and it is much the more easily managed of the two, besides being less liable to produce giddiness in those highly susceptible. Care must be exercised in galvanization, from the proximity of the basilar brain. Extremely interesting illustrations might be given in this connection did not brevity forbid.

Diet is all-important. It should be nutritious, but all veal and pork compounds must be tabooed, with all indigestible substances. Tobacco is undoubtedly highly injurious. The so-called grape-cure acts a good part, especially in those who can afford the expense of living at the vineyards.

Much more might be said and many particulars present their claims upon our attention, but your patience has been taxed already. Disagreeable as such patients are to handle, it is our duty to exert our utmost ability in their behalf, not only for their personal welfare, but for the comfort of their families and society at large. Other formidable maladies have succumbed to medical skill, and why not that under consideration? It lies within the power of gentlemen such as compose our society to do much in this direction, and assuredly the need is urgent.

Although I purposely refrained from touching the causation or pathology of post-nasal catarrh, one point which has interested me may be alluded to in closing, which is the belief in my own mind, from close observation, that under certain conditions the disease is contagious, even when positively non-syphilitic. I have repeatedly seen it occur in newly-married persons, and where, at school, children previously entirely healthy became affected when sleeping together. In all such instances noted no hereditary tendency existed, nor were other members of the family thus diseased. I have not heard this idea expressed by others, and many whom I have consulted dissent from it, yet through indubitable evidence I do not hesitate to assert my opinion, for if it is correct we should be on our guard, and in those thus exposed remember that "prevention is better than cure."—*Philadelphia Med. Times.*

